# Extensive serum biomarker analysis in the prethrombotic state of recurrent spontaneous abortion

**DOI:** 10.1111/jcmm.16671

**Published:** 2021-06-16

**Authors:** Ying Wu, Mingwei Xin, Qian Han, Jingshang Wang, Xiaodan Yin, Junqin He, Chenghong Yin

**Affiliations:** ^1^ Department of Traditional Chinese Medicine Beijing Obstetrics and Gynecology Hospital Capital Medical University Beijing China

**Keywords:** biomarker, prethrombotic state, Quantibody array, recurrent spontaneous abortion

## Abstract

The prethrombotic state (PTS) is a possible cause of recurrent spontaneous abortion (RSA). The aim of this study was to identify serum biomarkers for the detection of RSA with PTS (PSRSA). A Quantibody array 440 was used to screen novel serum‐based biomarkers for PSRSA/NRSA (RSA without PTS). Proteins differentially expressed in PSRSA were analysed using bioinformatics methods and subjected to a customized array and enzyme‐linked immunosorbent assay (ELISA) validation. We used receiver operating characteristic to calculate diagnostic accuracy, and machine learning methods to establish a biomarker model for evaluation of the identified targets. 20 targets were selected for validation using a customized array, and seven targets via ELISA. The decision tree model showed that IL‐24 was the first node and eotaxin‐3 was the second node distinguishing the PSRSA and NRSA groups (an accuracy rate of 100% and an AUC of 1). Epidermal growth factor (EGF) as the node distinguished the PSRSA and NC groups (an accuracy rate of 100% and an AUC of 1). EGF as the node distinguished the NRSA and NC groups (an accuracy rate of 96.5% and an AUC of 0.998). Serum DNAM‐1, BAFF, CNTF, LAG‐3, IL‐24, Eotaxin‐3 and EGF represent a panel of promising diagnostic biomarkers to detect the PSRSA.

## 1 INTRODUCTION

Recurrent spontaneous abortion (RSA) generally refers to the spontaneous loss of two or more pregnancies before 24 weeks of gestational age.^1^ About 1%‐5% of women of childbearing age experience recurrent miscarriage (spontaneous abortion).^2^ The causes of RSA are complex and diverse, usually lacking specific clinical manifestations. The known possible causes of RSA include genetic factors, uterine anomalies, endocrine anomalies, infectious diseases, immune dysfunction, prothrombotic state (PTS), systemic diseases and environmental causes during pregnancy.^3^, ^4^, ^5^

Prethrombotic state, also called thrombophilia, refers to a pathological dysfunction or disorder of the haemostatic, coagulation, anticoagulation and fibrinolytic system caused by multiple factors.^6^ PTS includes both inherited and acquired forms. Inherited PTS is comprised by a factor V Leiden acquired mutation (FVL), deficiency of antithrombin (AT) III, protein C (PC) or S (PS), histidine‐rich glycoprotein deficiency, prothrombin‐related thrombophilia, prothrombin 20210A mutation, elevated factor VIII level, and mutations of the gene encoding the enzyme methylenetetrahydrofolate reductase. Acquired PTS is comprised mainly by antiphospholipid antibody syndrome (APS) and various diseases or conditions, such as tumours and long‐term immobilization that cause blood hypercoagulability. The general prevalence of PTS in the general population is 3% ~ 8%, and in a study of women with recurrent spontaneous abortion, after excluding other causes, the prevalence of the PTS reached 78%.^7^ Hereditary PTS factors affect 3% to 11% of the population.^8^ Mitic et al^9^ reported that inherited thrombophilia was found in 36% of the study subjects. APS is an acquired autoimmune thrombotic disease, and RSA is one of its clinical classification criteria.^10^ Therefore, it is particularly important to study the atypical relationship between PTS and early spontaneous pregnancy loss.

According to the current PTS clinical testing protocols, RSA patients were divided into a PTS group and a non‐PTS group. Anticoagulant therapy was performed on the PTS group. Some patients had significant therapeutic effects, while some patients had insignificant therapeutic effects. It is necessary to find more accurate diagnostic markers to diagnose RSA patients with PTS in order to improve treatment.

Clinically, multi‐factor detection using protein chip technology can be used to screen and identify a panel of specific RSA protein markers and, combined with PTS detection technology, provides an important theoretical basis for the screening of RSA caused by PTS. Previously, Wu et al^11^ utilized a label‐based Human L1000 Array (Catalog #: AAH‐BLG‐1000; RayBiotech Inc.) to screen and identify four specific proteins, IGFBP‑rp1 / IGFBP‑7, Dkk3, angiopoietin‑2 and RAGE, which can be used to predict recurrent abortion.

Here, we employed a Quantibody^®^ Human Cytokine Antibody Array 440 (Catalog #: QAH‐CAA‐440, RayBiotech Inc) to screen and validate specific biomarkers for RSA with PTS (PSRSA) or RSA without PTS (NRSA) compared against women with normal pregnancy history (NC). Eighteen proteins were significantly differentially expressed in PSRSA serum compared to NRSA, including 11 up‐regulated and seven down‐regulated biomarkers. The custom array and ELISA results were consistent with the antibody array data. The serum levels of DNAM‐1, BAFF, CNTF, LAG‐3, IL‐24, Eotaxin‐3 and epidermal growth factor (EGF) provided high diagnostic accuracy in PSRSA, with area under the curve (AUC) values of 1.000, 1.000, 0.996, 0.982, 0.976, 0.972 and 0.862, respectively. Serum DNAM‐1, IL‐24, CNTF, LAG‐3, BAFF, Eotaxin‐3 and EGF represent a panel of promising diagnostic biomarkers to detect the prethrombotic state in RSA.

## 2 MATERIALS AND METHODS

### 2.1 Subjects

One sixty‐five subjects were recruited from the Department of Traditional Chinese Medicine, Beijing Obstetrics and Gynecology Hospital, Capital Medical University (Beijing, China). All subjects signed informed consent forms before participating in this study. This study was approved by the Ethics Committee of the Beijing Obstetrics and Gynecology Hospital, Capital Medical University (approval number: 2016‐KY‐001). None of the subjects in this study (<40 years of age) were in a gestational state. All samples were collected during the subjects' menstrual cycles. Studies have shown that the menstrual cycle will not affect the results of these experiments.^12^ Patient endocrine levels were normal and their partners' sperm function was normal. They had no serious diseases, such as abnormal liver function, abnormal kidney function or cardio‐cerebrovascular disease. Serum samples were collected and stored at −80℃ for use.

The diagnostic criteria for PTS were pre‐diffusive intravascular coagulation (pre‐DIC), the Diagnostic Reference Standards Developed by the Seventh National Conference on Thrombosis and Hemostasis^13^ and ‘Thrombosis & Hemostasis‐Basic Principles & Clinical Practice’.^14^ Currently, commonly used molecular markers include antithrombin III activity (AT‐Ⅲ), protein C activity (PC), protein S activity assay (PS), plasminogen activity (PLG), tissue‐type plasminogen activator (t‐PA), plasminogen activator inhibitor (PAI‐1), fibrinogen degradation product (FDP), CD3‐CD16 + CD56 + and homocysteine (HCY).

All RSA patients met the diagnostic criteria for RSA and had pregnancy losses prior to 12 weeks. The RSA patients who had one or more atypical PTS diagnostic markers were deemed PSRSA. The RSA patients who did not have one or more atypical PTS diagnostic markers were deemed NRSA. Women with normal pregnant history were deemed NC as the healthy controls, and they did not meet the diagnostic criteria for PTS.

According to the diagnostic criteria for RSA and PTS, the subjects were divided into 58 PSRSA, 55 NRSA and 52 NC subjects.

### 2.2 Quantibody array

Twenty four PSRSA, 12 NRSA and 12 NC subjects were studied in the biomarker discovery stage. The characteristics, including number of subjects, age of diagnosis, body mass index (BMI) and time of spontaneous abortion, are shown in Table 1. The differences between groups were analysed by Welch's *t* test, and *P* values <.05 were considered statistically significant. The Quantibody array QAH‐CAA‐440 was used to measure the serum protein levels, according to the manufacturer's instructions. It is an array‐based multiple ELISA system, which can simultaneously and quantitatively detect the expression levels of 440 proteins and was employed as described previously.^11^ Briefly, the serum samples were diluted 1:2, added to a chip coated with capture antibodies, and incubated overnight at 4℃. The chip was washed and incubated with a biotin‐labelled secondary antibody for 2 hours at room temperature. The chip was washed again, and Cy3‐conjugated streptavidin was added to the chip. Fluorescent signal was measured with an InnoScan 300 Microarray Scanner (Innopsys). The signal values were extracted using Mapix software. The data were normalized against positive controls.

**TABLE 1:** Participant characteristics in the biomarker discovery stage

Variable	Normal reference value (Unit)	PSRSA	NRSA	NC	PSRSA‐NRSA *P* value (Welch's *t* test)	PSRSA‐NC *P* value (Welch's *t* test)	NRSA‐NC *P* value (Welch's *t* test)
Number	NA	24	12	12	NA	NA	NA
Age at diagnosis	NA	31.83 ± 4.91	31.75 ± 6.11	30.85 ± 4.87	.9677	.6008	.7154
BMI	NA	22.36 ± 2.32	21.17 ± 2.83	20.93 ± 3.04	.2218	.1685	.8442
Timing of spontaneous abortions	NA	2.54 ± 1.25	2.83 ± 1.34	0	.5354	<.0001	<.0001
AT‐III	80‐120 (%)	117.92 ± 15.00	111.75 ± 6.84	101.67 ± 8.11	.0997	.0002	.0034
PC	70‐140 (%)	95.65 ± 30.36	103.76 ± 17.50	103.78 ± 17.67	.3179	.3189	.9984
PS	60‐130 (%)	65.83 ± 34.17	82.96 ± 17.82	100.33 ± 14.75	.0563	.0002	.0166
PLG	80‐150 (%)	106.79 ± 23.15	103.58 ± 7.25	103.67 ± 8.21	.5394	.5585	.9792
t‐PA	1.0‐12.0 (ng/mL)	2.39 ± 1.83	1.91 ± 0.81	3.11 ± 1.23	.2767	.1733	.0106
PAI‐1	5‐45 (ng/mL)	30.39 ± 16.25	25.17 ± 10.11	29.11 ± 8.05	.2468	.7545	.3036
FDP	0‐5.0 (µg/mL)	<2.5	<2.5	<2.5	NA	NA	NA
CD3‐CD16 +CD56+	9.5‐23.5 (%)	13.56 ± 7.82	12.89 ± 7.17	13.87 ± 3.87	.8030	.8763	.6838
HCY	0‐15 (µmol/L)	8.17 ± 3.04	8.58 ± 2.75	7.12 ± 3.25	.6915	.3594	.2481

Abbreviations: AT‐III, antithrombin‐Ⅲ; BMI, body mass index; FDPs, fibrinogen degradation products; HCY, homocysteine; PAI‐1, plasminogen activator inhibitor 1; PC, protein C; PLG, plasminogen; PS, protein S; t‐PA, tissue plasminogen activator.

### 2.3 Bioinformatics analysis

All array data analyses were performed using RayBio Analysis Tool software (Q‐Analyzer Software for QAH‐CAA‐440) (https://www.raybiotech.com/products/other‐products/software/). To elucidate the potential functions of the differentially expressed proteins obtained from the Quantibody array, gene ontology (GO) and pathway analysis were carried out to describe the biological processes, cellular components and molecular functions of these proteins by inputting the gene IDs of the differential proteins into the KOBAS3.0 database (http://kobas.cbi.pku.edu.cn/index.php). Protein‐protein interaction (PPI) analysis was performed using the STRING database (https://string‐db.org/cgi/input.pl) and the protein IDs to identify node proteins.

### 2.4 Custom Array for 20 targets (CQ20) and ELISA

Twenty proteins identified by QAH‐CAA‐440 were selected for evaluation by a special Custom Antibody Array (CQ20: Human Custom Antibody, RayBiotech Inc) using 117 subjects (58 PSRSA, 34 NRSA and 25 NC, including the 48 subjects studied in biomarker discovery stage). The serum levels of 20 proteins were measured, following the manufacturer's instructions.

Seven proteins, DNAM‐1, IL‐24, CNTF, LAG‐3, BAFF, Eotaxin‐3 and EGF, were selected for ELISA validation. Seven ELISA kits were purchased from RayBiotech, Inc. For improving the accuracy of ELISA validation, the number of subjects was increased to 165. ELISA assays were performed according to the manufacturer's instructions. The characteristics of the 165 subjects, including number of subjects, age of diagnosis, BMI and time of spontaneous abortion, are shown in Table 2. The differences between groups were analysed by Welch's *t* test, and *P* values <.05 were considered statistically significant. Briefly, the serum samples were diluted (1:2), added to a plate coated with capture antibody and incubated overnight at 4℃. The plate was washed and incubated with a biotin‐binding antibody (1:5000) for two h at room temperature. The plate was washed again and HRP‐conjugated streptavidin (1:1000) and TMB substrate were added and allowed to incubate for 30 min at room temperature. OD_450_ values were measured using an ELx800NB plate reader (Bio‐Tek, Inc).

**TABLE 2:** Participant characteristics in the biomarker validation stage

Variable	Normal reference value (Unit)	PSRSA	NRSA	NC	PSRSA‐NRSA *P* value (Welch's *t* test)	PSRSA‐NC *P* value (Welch's *t* test)	NRSA‐NC *P* value (Welch's *t* test)
Number	NA	58	55	52	NA	NA	NA
Age at diagnosis	NA	31.59 ± 4.82	31.64 ± 4.30	31.00 ± 4.72	.9535	.5071	.4544
BMI	NA	21.89 ± 2.63	21.05 ± 2.50	21.03 ± 2.46	.0903	.0796	.9122
Timing of spontaneous abortions	NA	2.38 ± 1.02	2.40 ± 0.85	0	.9070	<.0001	<.0001
AT‐III	80‐120 (%)	114.72 ± 12.33	108.64 ± 7.88	104.18 ± 7.76	.0022	<.0001	.0013
PC	70‐140 (%)	99.64 ± 29.37	103.57 ± 15.77	105.79 ± 19.13	.3750	.2234	.5997
PS	60‐130 (%)	67.00 ± 31.69	94.83 ± 17.05	102.67 ± 16.33	<.0001	<.0001	.0240
PLG	80‐150 (%)	103.21 ± 23.00	104.91 ± 11.57	10.53 ± 8.56	.6178	.5977	.9943
t‐PA	1.0‐12.0 (ng/mL)	2.85 ± 2.63	3.73 ± 2.41	4.03 ± 1.48	.0666	.0182	.8242
PAI‐1	5‐45 (ng/mL)	25.41 ± 13.69	25.19 ± 8.26	25.14 ± 8.02	.9159	.7618	.5848
FDP	0‐5.0 (µg/mL)	<2.5	<2.5	<2.5	NA	NA	NA
CD3‐CD16 + CD56+	9.5‐23.5 (%)	13.24 ± 7.15	14.17 ± 8.78	14.32 ± 4.10	.5448	.3863	.9742
HCY	0‐15 (µmol/L)	9.46 ± 3.93	7.99 ± 3.14	7.39 ± 3.99	.0367	.0047	.3468

Abbreviations: AT‐III, antithrombin‐Ⅲ; BMI, body mass index; FDPs, fibrinogen degradation products; HCY, homocysteine; PAI‐1, plasminogen activator inhibitor 1; PC, protein C; PLG, plasminogen; PS, protein S; t‐PA, Tissue plasminogen activator.

### 2.5 Statistical analysis

Data were presented as means ± SD (standard deviations). Differences between groups were determined by one‐way ANOVA, followed by multiple comparisons performed with the post hoc Bonferroni test (SPSS version 20; IBM Corp.). *P* values <.05 were considered statistically significant. Fold change (FC) was calculated to indicate the relative expression levels of proteins in PSRSA patient serum compared to NRSA and NC. Data mining cluster analysis was used to identify potential biomarkers by clustering all relevant proteins according to the similarity of their expression profiles using Cluster software version 3.0 (http://cluster2.software.informer.com/3.0). The receiver operating characteristics curve (ROC) method was used to assess sensitivity and specificity of potential biomarkers. A decision tree model was built using the R language machine software package C5.0 algorithm.^15^

## 3 RESULTS

### 3.1 Study population characteristics

We recruited a total of 165 subjects, 48 subjects in the biomarker discovery stage and all 165 subjects in the biomarker validation stage. According to the diagnostic criteria for PTS, combined with the literature, the RSA patients with prethrombotic state were diagnosed with at least one abnormal indicator as follows: antithrombin III activity (AT‐III), protein C activity (PC), protein S activity determination (PS), plasminogen activity (PLG), tissue‐type plasminogen activator (t‐PA), plasminogen activator inhibitor (PAI‐1), plasmin degradation products (FDP), CD3‐CD16 + CD56 + and/or homocysteine.

A case‐control study was performed between 52 women NC subjects and 113 RSA patients. The RSA patients were divided into two groups: the PSRSA group with 58 patients and the NRSA group with 55 patients. Overall, 24 PSRSA, 12 NRSA patients and 12 NC subjects (Table 1) were included in the cytokine profile screening using the Quantibody array QAH‐CAA‐440. A total of 117 serum samples, 58 PSRSA, 34 NRSA patients and 25 NC subjects, were analysed using Custom Arrays CQ20 for validation. A total of 165 subjects (Table 2) were employed for ELISA validation. The demographic data of the participants including the number, age, BMI, history of RSA and the detection index are summarized in Tables 1 and 2.

Table 1 shows that there were no significant differences in the indicators between PSRSA and NRSA. The AT‐III and PS indicators were significantly different (*P* < .05) between PSRSA and NC. The AT‐III, PS, and t‐PA indicators were significantly different (*P* < .05) between NRSA and NC. As the sample size increased, the differences in indicators between PSRSA and NRSA appeared. Table 2 shows that the AT‐III, PS and HCY indicators had significant differences (*P* < .05) between PSRSA and NRSA. The AT‐III, PS, t‐PA and HCY were significantly different (*P* < .05) between PSRSA and NC. The AT‐III and PS indicators showed significant differences (*P* < .05) between NRSA and NC. Combining Tables 1 and 2, AT‐III and PS were able to distinguish the PSRSA, NRSA and NC groups.

### 3.2 Cytokine profiles in PSRSA vs NRSA or NC

**FIGURE 1:**
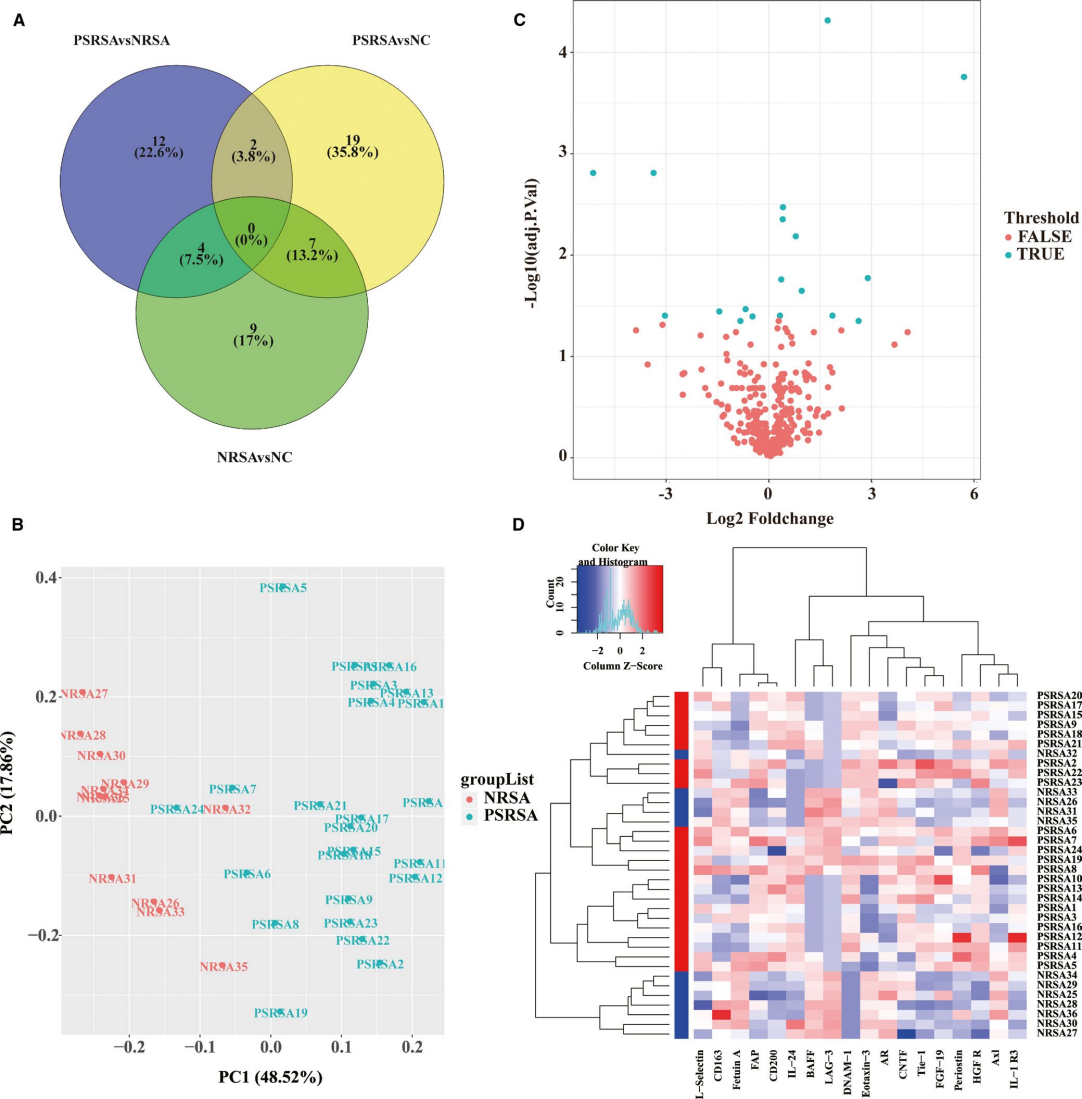
PSRSA specific biomarker analysis. (A) Venn diagram analysis. The proteins expressed differentially between the PSRSA, NRSA and NC groups were analysed by Venn diagram to identify PSRSA specific biomarkers. The blue circle presents PSRSA vs NRSA, the yellow circle presents PSRSA vs NC, the green circle presents NRSA vs NC. (B) Principal component analysis of PSRSA and NRSA. The PSRSA samples (blue) are sharply discriminated from the NRSA samples (red) on the second principal component (PC2) that accounts for 48.52% of all variability. (C) Volcano plot of candidate protein expression in PSRSA and NRSA from the Human Antibody Array QAH‐CAA‐440. Visualizing the proteins separated according to their log FC (*x*‐axis, FC: fold change) and significance (*y*‐axis: −log10 adjusted *P*. Val) in the PSRSA and NPSA groups. Proteins highlighted in blue had adjusted *P*. Val <.05 and fold change >1.2 or <0.83 in the data set. (D) Hierarchical clustering heatmap of proteins in PSRSA and NRSA. The levels of proteins are depicted as colours ranging from blue to white to red, presenting low, intermediate, and high concentration, respectively, according to the mean of each protein. The group of samples is shown on the right and by the coloured bar on the left (red represents PSRSA and blue represents NRSA)

A 440‐human cytokine antibody was used to quantitatively measure the cytokine profiles in 12 NC subjects, 12 NRSA subject and 24 PSRSA patients. For a single protein, a signal intensity fold change of ≥1.5 was defined as up‐regulated and ≤0.67‐fold as down‐regulated (*P* < .05). To identify biomarkers specific to PSRSA, the differentially expressed proteins were analysed statistically using one‐way ANOVA, followed by multiple comparisons performed with post hoc Bonferroni test between any pair of the PSRSA, NRSA and NC groups. As a result, 18 proteins were found to be differentially expressed between the PSRSA and NRSA groups (Table 3), 20 between the PSRSA and NC groups, and 21 between the NRSA and NC groups (detailed data in Tables [Link], [Link], [Link]). Therefore, after Venn diagram analysis, we identified 12 specific PSRSA associated biomarkers (Figure 1A).

**TABLE 3:** Cytokines differentially expressed between the PSRSA and NRSA groups

Protein ID	Gene ID	AveExp. PSRSA	AveExp. NRSA	LogFC	*P* value	adj. *P*. Val	Fold change	Regulation
DNAM‐1	10666	8.31	2.66	5.66	0	0	50.51	Up
IL‐24	11009	4.3	1.46	2.84	0	.02	7.18	Up
Periostin	10631	12.01	9.43	2.58	0	.05	5.96	Up
CNTF	1270	7.76	5.95	1.81	0	.04	3.51	Up
Tie‐1	7075	7.84	6.17	1.67	0	0	3.19	Up
IL‐1 R3	3556	10.06	9.15	0.91	0	.02	1.88	Up
FGF‐19	9965	8.32	7.58	0.74	0	.01	1.67	Up
FAP	2191	12.91	12.54	0.37	0	0	1.29	Up
HGF R	4233	11.26	10.91	0.36	0	0	1.28	Up
L‐Selectin	6402	15.42	15.1	0.31	0	.02	1.24	Up
CD200	4345	13.17	12.89	0.28	0	.04	1.21	Up
CD163	9332	16.99	17.51	−0.52	0	.04	0.69	Down
Axl	558	8.8	9.53	−0.73	0	.04	0.6	Down
Fetuin A	197	13.32	14.2	−0.88	0	.05	0.54	Down
AR	374	5.26	6.75	−1.5	0	.04	0.35	Down
Eotaxin‐3	10344	4.93	8.01	−3.09	0	.04	0.12	Down
BAFF	10673	1.82	5.23	−3.42	0	0	0.09	Down
LAG‐3	3902	1.31	6.5	−5.18	0	0	0.03	Down
EGF	1950	5.4	6.02	−0.62	.04	.21	0.65	Down

This analysis was confirmed using principal component analysis (PCA). PCA data showed that the PSRSA group had a distinct component of different proteins when compared to the NRSA and NC groups. Using the first two principal components of 18 differentially expressed proteins (DEPs), all 36 samples were clearly divided into two clusters according to their disease states (Figure 1B, Figures S1A and S2A). The distribution of these 18 proteins in the volcano plot is shown in Figure 1C, Figures S1B and S2B. The volcano plot shows 18 proteins with differential expression based on an adjusted *P* value (adj. *P*. Val) of less than .05. Of these, 11 proteins were up‐regulated, and seven proteins were down‐regulated (Figure 1C). To determine whether these DEPs could discriminate patients with PSRSA from the NRSA or NC groups, we performed the heatmap of hierarchical clustering analysis (Figure 1D, Figures S1C and S2C). The result showed that most of the PSRSA samples could be separated from the NRSA group to form two major groups, and the two clusters isolated through the different expression of proteins (Figure 1D).

### 3.3 Expression characteristics of PSRSA related proteins

**FIGURE 2:**
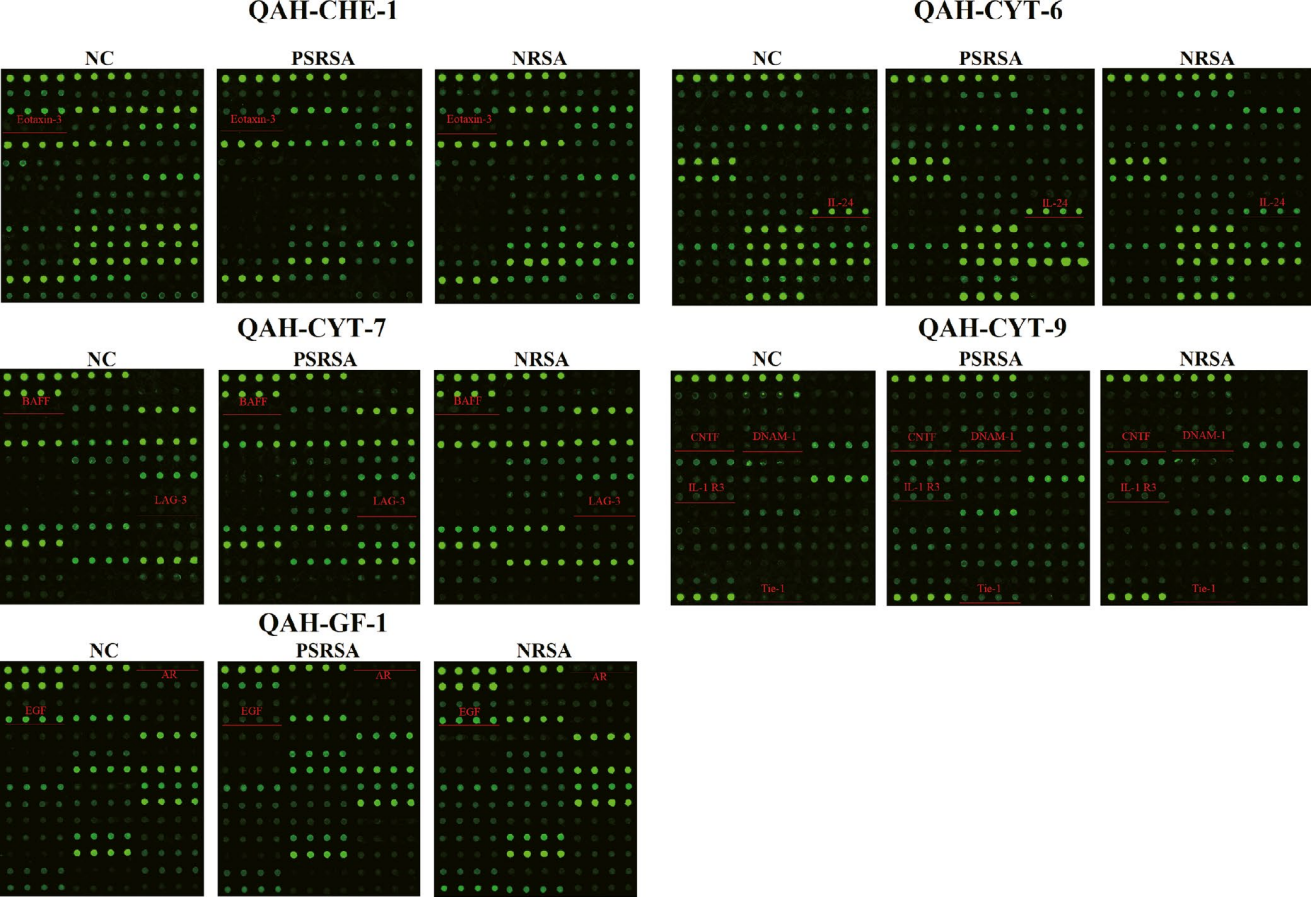
PSRSA patient serum biomarker profiles. In the profiles of QAH‐CAA‐440 antibody arrays, the levels of proteins were proportional to their fluorescence intensity. In these arrays, each antibody was printed in four duplicates, and the locations of the serum PSRSA patient biomarkers are noted in coloured lines

**FIGURE 3:**
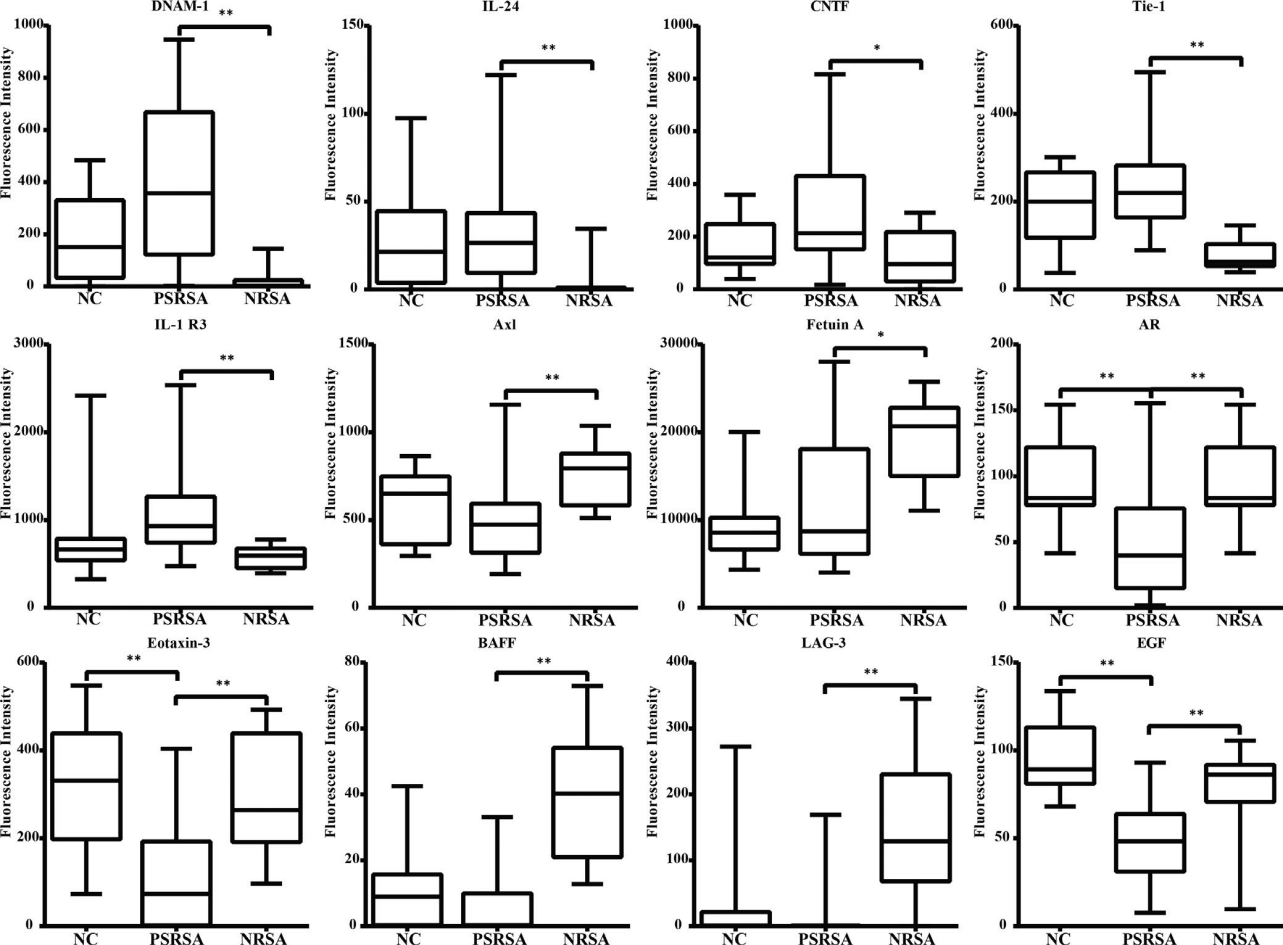
Boxplot of antibody array analysis. Based on the Venn analysis, 12 differentially expressed proteins were identified and are shown by boxplot from the PSRSA, NRSA and NC groups. The centre line in each boxplot presents the mean of data in each group. **P < .01 and *P < .05: PSRSA vs NRSA or NC

There were 18 proteins significantly differentially expressed in PSRSA patients as shown in Table 3 (detailed data of 440 factors are shown in Table [Link], [Link], [Link]). In the antibody arrays, the protein levels were proportional to their fluorescence intensity (Figure 2). The up‐regulated factors included DNAM‐1, IL‐24, Periostin, CNTF, Tie‐1, IL‐1 R3, FGF‐19, FAP, HGF R, L‐Selectin and CD200, DNAM‐1 being the most significant DEP. The down‐regulated proteins included CD163, Axl, Fetuin A, AR, Eotaxin‐3, BAFF and LAG‐3, with LAG‐3 being the most significant. The levels of proteins were further analysed by boxplot using the signal values. As shown in Figure 3, DNAM‐1, IL‐24, CNTF, Tie‐1, and IL‐1 R3 were increased, while AR, Eotaxin‐3, BAFF, LAG‐3 and EGF were decreased in the PSRSA group, when compared to the NRSA and NC groups.

### 3.4 Bioinformatics analysis

**FIGURE 4:**
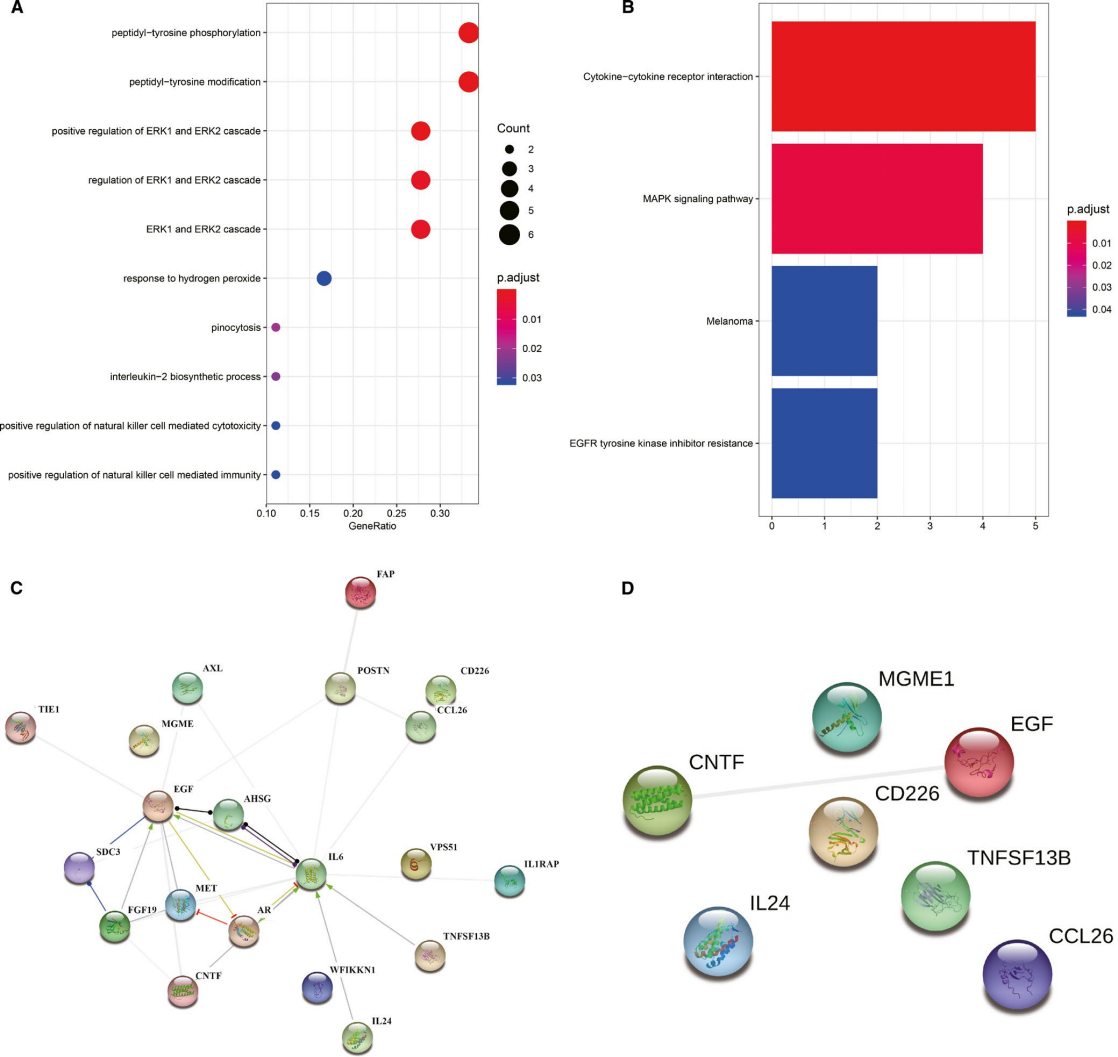
Bioinformatics analysis. (A) Gene Ontology terms associated with the biological processes. Statistical significance of the GO terms was established by Fishers Exact test. GO terms with differential genes ≥5 *P* values <.05 were considered statistically significant. (B) KEGG pathway analysis indicates the significant pathways enriched by differentially expressed proteins in PSRSA patients. The top 4 pathways for the differentially expressed proteins are displayed. The *x*‐axis shows the amounts of proteins associated with the KEGG pathway. The colour ranging from blue to red presents different levels of adjusted *P* value. (C) and (D) Protein‐protein interaction (PPI) networks. PPI analysis was performed for the 20 differentially expressed proteins (C) and 7 specific proteins (D). The line between two proteins means that there is correlation with these two proteins in the biological function and the line thickness indicates the strength of data support

There were 30 statistically significant BP items annotated (*P* < .05), 2 statistically significant CC items (*P* < .05) and 28 statistically significant MF items (*P* < .05) (Table S4). DEPs (PSRSA vs NRSA) were involved in peptidyl‐tyrosine phosphorylation, peptidyl‐tyrosine modification, positive regulation of ERK1 and ERK2 cascade, regulation of ERK1 and ERK2 cascade, ERK1 and ERK2 cascade, response to hydrogen peroxide, pinocytosis and interleukin‐2 biosynthetic process. Multiple biological processes, such as positive regulation of natural killer cell‐mediated cytotoxicity and positive regulation of natural killer cell‐mediated immunity, were identified. The 10 DEPs with the highest credibility were filtered according to their *P* value, and a bubble chart (Figure 4A) was drawn. In the KEGG pathway analysis, a total of 4 KEGG pathways with statistical significance (*P* < .05) were annotated. The biologically regulated pathways with significantly different changes in PSRSA vs NRSA involved cytokine‐cytokine receptor interaction, MAPK signalling pathway, melanoma and EGFR tyrosine kinase inhibitor resistance (Figure 4B). Figure 4C,D show the interactions of these differentially expressed proteins and EGF interactions with CNTF, FGF‐19, Tie‐1, AR, IL‐6 and SDC3.

### 3.5 CQ20 validation

**FIGURE 5:**
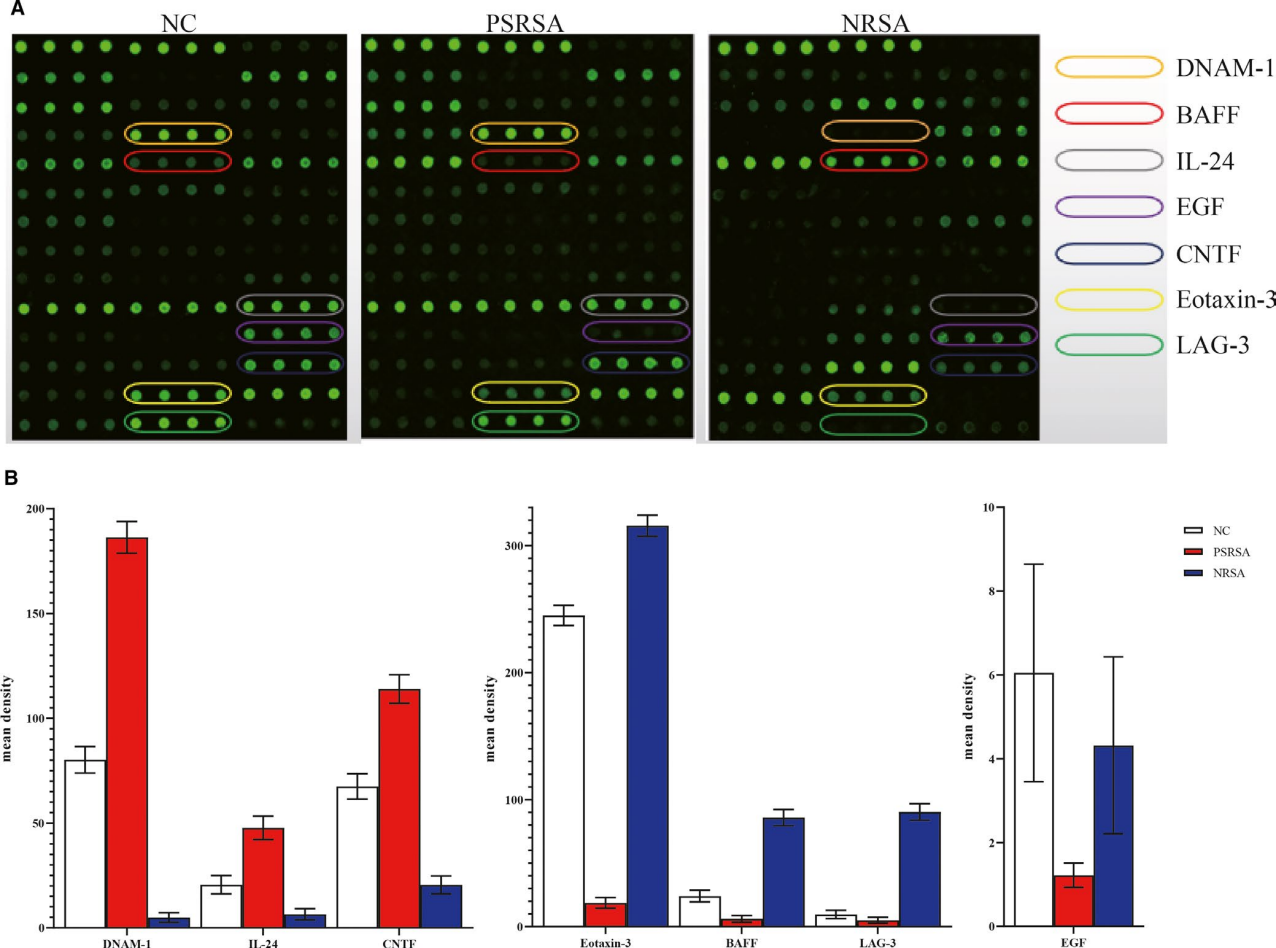
Validation of serum PSRSA patient proteins by the customized array. (A) The fluorescence intensity in the profiles of the customized arrays. The levels of proteins are proportional to their fluorescence intensity. In this array, each antibody was printed in four duplicates, and the locations of the serum proteins in the NC, PSRSA and NRSA groups are noted in coloured boxes. (B) Histogram of customized array data.^7^ differentially expressed proteins are shown by histogram in the PSRSA, NRSA and NC groups. *P < .05 vs NRSA or NC groups

Using Venn graph intersection analysis to identify PSRSA and NRSA or NC common differential factors, DNAM‐1, IL‐24, Periostin, CNTF, Tie‐1, IL‐1 R3, FGF‐19, FAP, HGF R, L‐Selectin, CD200, CD163, Axl, Fetuin A, AR, Eotaxin‐3, BAFF and LAG‐3 were selected. Combined with their clinical significance, the factors L‐Selectin, CD200 and CD163 were excluded, and Syndecan‐3, ANG‐2, IL‐6, GASP‐2 and EGF were added. Finally, 20 proteins of interest were selected to customize the array (Table 4). 58 PSRSA, 34 NRSA and 25 NC cases were recruited to verify the 20 proteins. Table 4 shows the differential expression data of the 20 proteins from the customized array in the PSRSA, NRSA and NC groups. Compared with the NRSA and NC groups, the expressions of DNAM‐1, IL‐24, CNTF, Tie‐1, Periostin, Syndecan‐3, ANG‐2, FGF‐19, IL‐1 R3, FAP and HGF R were up‐regulated in the PSRSA group, while Fetuin A, Axl, IL‐6, AR, Eotaxin‐3, GASP‐2, BAFF, LAG‐3 and EGF were down‐regulated, consistent with the primary screening results. DNAM‐1, IL‐24, CNTF, Eotaxin‐3, BAFF, LAG‐3 and EGF were the most significantly differentially expressed in the PSRSA group, compared against the NRSA and NC groups (Figure 5A,B).

**TABLE 4:** Twenty specific PSRSA biomarkers in the custom array

Protein ID	Gene ID	PSRSA vs NC		NRSA vs NC		PSRSA vs NRSA	
		Fold change	*P* value	Fold change	*P* value	Fold change	*P* value
DNAM‐1	10666	2.323	.004	0.062	.629	37.687	.000
IL‐24	11009	2.323	.091	0.314	.079	7.400	.016
CNTF	1270	1.689	.228	0.303	.002	5.566	.039
Tie‐1	7075	1.267	.202	0.315	.052	4.025	.000
Periostin	10631	3.139	.538	0.416	.000	7.548	.041
Syndecan‐3	9672	2.323	.001	1.117	.780	2.080	.001
ANG‐2	285	1.414	.117	0.662	.014	2.136	.015
FGF‐19	9965	1.430	.204	0.890	.001	1.606	.007
IL‐1 R3	3556	2.480	.000	1.677	.974	1.479	.022
FAP	2191	1.495	.003	0.939	.280	1.592	.004
HGF R	4233	1.780	.028	1.477	.316	1.205	.261
Fetuin A	197	1.321	.505	1.388	.007	0.951	.280
Axl	558	0.707	.148	1.369	.004	0.516	.000
IL‐6	3569	0.856	.034	1.582	.340	0.541	.122
AR	374	0.588	.090	1.658	.000	0.354	.000
Eotaxin‐3	10344	0.076	.214	1.288	.388	0.059	.003
GASP‐2	100528062	4.258	.000	63.629	.108	0.067	.058
BAFF	10673	0.258	.034	3.567	.000	0.072	.002
LAG‐3	3902	0.528	.489	9.399	.090	0.056	.002
EGF	1950	0.202	.045	0.714	.415	0.283	.000

### 3.6 Validation array results with ELISA

**FIGURE 6:**
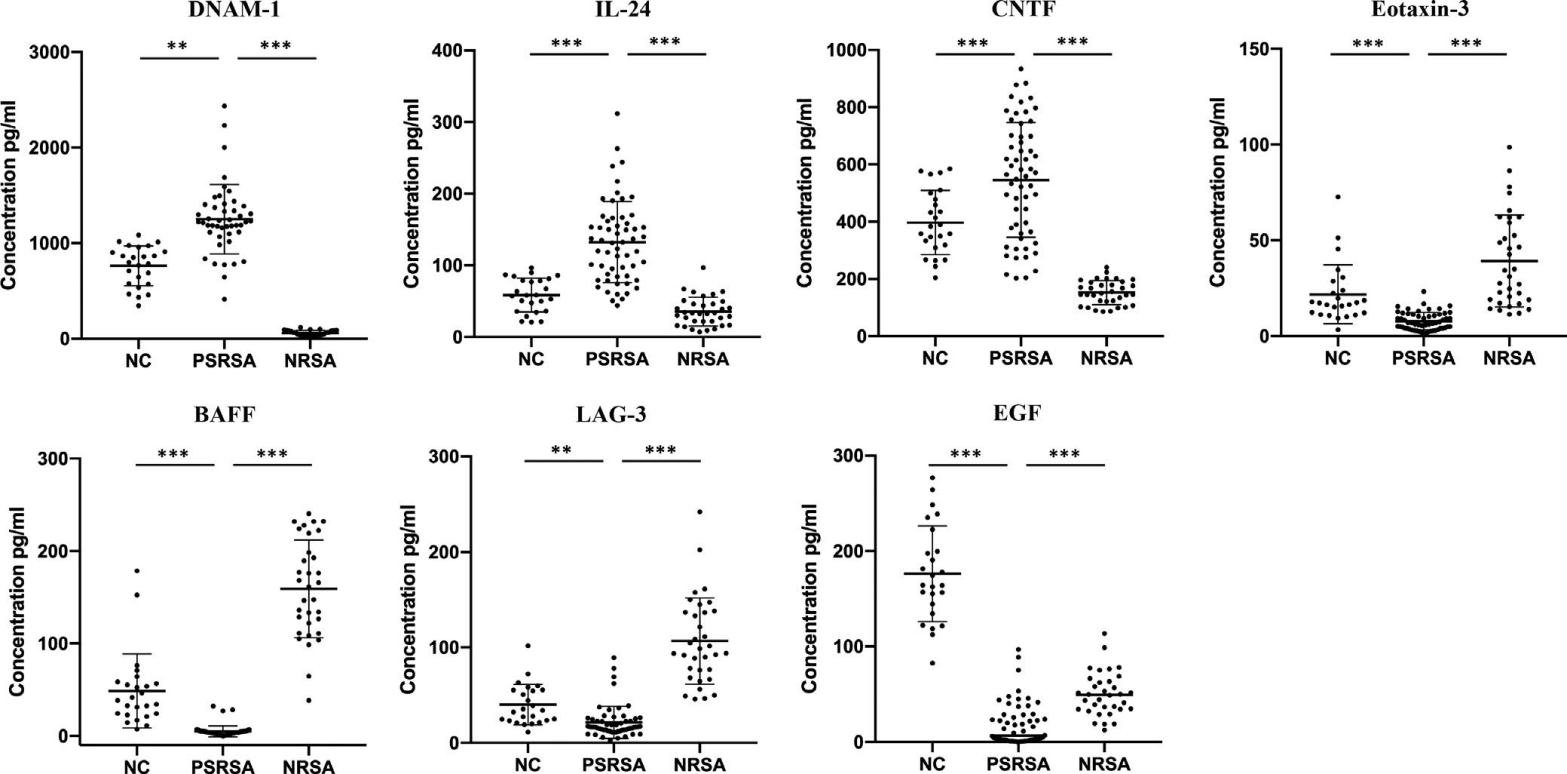
ELISA validation results of serum PSRSA biomarkers. The data is shown in the scatter plot with median values. The *P* value between PSRSA vs NRSA or NC of each protein was obtained from the Mann‐Whitney *U* test analysis. ***P < .0001, **P < .001 and *P < .05 vs NRSA or NC groups

According to the primary screening and the customized array results, 7 proteins of interest were selected for further validation using ELISA on samples from 165 subjects. As shown in Figure 6, the levels of DNAM‐1, IL‐24 and CNTF were increased in PSRSA patients, compared with the NRSA and NC groups. The levels of Eotaxin‐3, BAFF, LAG‐3 and EGF were decreased in the PSRSA group, compared with the NRSA and NC groups. These results were consistent with the array results.

### 3.7 Sensitive and specific analysis of several biomarkers

**FIGURE 7:**
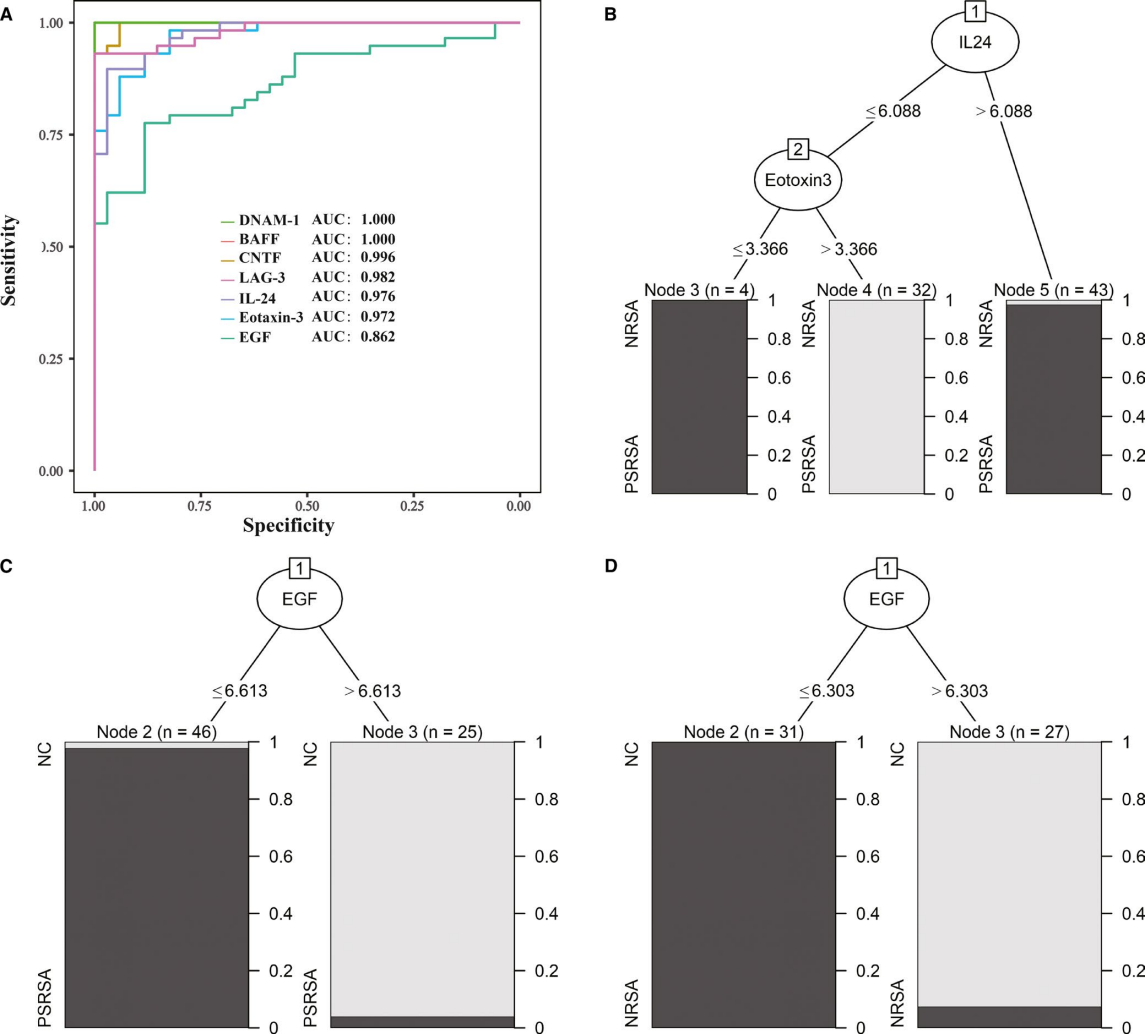
Statistical analysis. (A) The ROC curve analysis for 7 proteins differentially expressed among the PSRSA, NRSA and NC groups. The results were obtained from the biomarker decision tree model: (B) PSRSA vs NRSA decision tree model, IL‐24 as the first node and eotaxin‐3 as the second node distinguished the PSRSA and NRSA groups. (C) PSRSA vs NC decision tree model. Epidermal growth factor (EGF) as the node distinguished the PSRSA and NC groups. (D) NRSA vs NC decision tree model. EGF as the node could distinguished the NRSA and NC groups

We used one‐way ANOVA, followed by multiple comparisons performed with post hoc Bonferroni test, to analyse the differences between PSRSA and NRSA or NC in order to identify markers specific to PSRSA. The ROC curves of these 7 proteins were used to analyse the sensitivity and specificity in PSRSA patients. The AUC values were 1.000 (DNAM‐1), 1.000 (BAFF), 0.996 (CNTF), 0.982 (LAG‐3), 0.976 (IL‐24), 0.972 (Eotaxin‐3) and 0.862 (EGF) (Figure 7A). DNAM‐1 and BAFF had the highest sensitivity, specificity and AUC, followed by CNTF, LAG‐3, IL‐24, Eotaxin‐3 and EGF. Moreover, the combination of ROC, DNAM‐1 and BAFF showed the greatest diagnostic performance for distinguishing PSRSA and NRSA, with an AUC of 1.000.

The decision tree model showed that IL‐24, Eotaxin‐3 and EGF were part of the model (Figure 7B‐D). The model distinguished the PSRSA and NRSA groups, with IL‐24 as the first node and Eotaxin‐3 as the second node, with a rate of 100% (sensitivity 100% and specificity 100%), a Kappa coefficient of 1 and a positive predictive value of 1. The negative predictive value was 1, and the AUC was 1. This showed that, when comparing PSRSA and NRSA, the combination of IL‐24 and Eotaxin‐3 achieved the greatest discrimination effect (Figure 7B). Using EGF as the node to distinguish PSRSA from the NC groups, the model accuracy rate was 100% (sensitivity 100%, specificity 100%), the Kappa coefficient was 1, the positive predictive value and negative predictive value were 1, and the AUC was 1 (Figure 7C). Interestingly, when using EGF as the node to distinguish the NRSA and NC groups, the model accuracy rate was 96.5% (sensitivity 100%, specificity 93.94%), the Kappa coefficient was 0.9304, the positive predictive value was 0.9259, the negative predictive value was 1, and the AUC was 0.998 (Figure 7D), indicating that the EGF indicator distinguished the two groups with the greatest effect.

## 4 DISCUSSION

As one of the most prevalent obstetric complications, recurrent spontaneous abortion affects more than 30% of pregnancies.^16^ Although RSA has been the focus of the majority of research, in most cases the cause of RSA is not clear. Recently, the relationship between RSA and the prethrombotic state has become a focus of attention.^17^ The prethrombotic state causes decidual vascular fibrinoid necrosis and villus infarction, which affects the material exchange between the foetus and mother, increasing the risk of pregnancy loss.^18^ Thrombus formation occurs as a result of the combined effects of changes in various factors such as vascular endothelial cells, platelets, coagulation, anticoagulation, fibrinolytic system, and haemorheology. These factors have changed to varying degrees prior to thrombosis.^19^ Therefore, it is particularly important to find methods for early screening and diagnosis of PTS, when appropriate measures can be taken to intervene and to prevent recurrent spontaneous abortion. Eighteen meaningful proteins were initially identified for further verification. The seven proteins DNAM‐1, IL‐24, CNTF, LAG‐3, BAFF, Eotaxin‐3 and EGF were completely consistent with the initial screening results. These distinctions could be used as a specific marker of RSA caused by thrombosis. In addition, the identified seven proteins were used to build decision trees, and IL‐24, Eotaxin‐3 and EGF were confirmed as classification models for PSRSA vs NRSA or NC. The purpose was to evaluate and predict the prevalence of RSA and provide a reference to clinicians to better evaluate treatment options.

### 4.1 Epidermal growth factor

Epidermal growth factor is a multifunctional growth factor. Many studies have shown that the survival and invasion capabilities of human trophoblast cells are related to the intercellular signalling of EGF‐related peptides.^20^ EGF can reduce trophoblast apoptosis induced by exposure to oxidative stress in an in vitro experiment.^21^ Yumusak et al^22^ found that EGF was effective in organization of the thrombus by accelerating healing in the thrombosed vessel wall and triggering neovascularisation in an experimental rat model. However in the literature, there are few reports concerning the effect of EGF on human PTS. In our study, compared with the NRSA group, the serum levels of EGF were decreased in PSRSA patients. The relationship between EGF and PSRSA requires further investigation.

### 4.2 DNAX accessory molecule‐1

DNAX accessory molecule‐1 (DNAM‐1) is also known as platelet / T cell activation antigen 1 (PTS1),^23^ also designated as CD226.^24^ DNAM‐1 is involved in the differentiation of cytotoxic T lymphocytes and anomalous killer cells,^25^ is expressed on platelets and is involved in platelet activation and aggregation.^23^ It is also involved in intercellular injection and lymphocyte signal transfer and is widely expressed on T cells, NK cells, monocytes and B cells, among other types of leukocytes.^26^ Xu et al^27^ found that CD226 might be involved in complications of vascular endothelial cells and platelets, suggesting that CD226 and PDGF may be involved in the vascular immune injury of pregnancy‐induced hypertension, which in turn triggers the related mechanism of intraluminal microthrombosis. Perricone et al^28^ found that the number and proportion of NK cells in the peripheral blood of RSA patients with APS were significantly higher than those of non‐RSA patients with APS, and the numbers of NK cells might be related to the decrease in gestational age in APS‐RSA patients during pregnancy loss, forming the hypothesis that NK cells promote the development of RSA in APS patients. Taken together, DNAM‐1 participates in platelet activation, aggregation, adhesion to promote the formation of thrombus, and activation of NK cells, and may promote NK cells to produce cytotoxicity.

### 4.3 Interleukin‐24

Interleukin‐24 (IL‐24) is a secreted cytokine that belongs to the IL‐10 cytokine family. It is also known as melanoma differentiation associated gene‐7 (MDA‐7). Shao et al^29^ found that human‐foetal interface decidual stromal cells express IL‐24 and its receptors. IL‐24 significantly inhibited the viability of decidual stromal cells and promoted their apoptosis, while anti‐IL‐24 and IL‐22R1 neutralizing antibody significantly promoted the growth of decidual stromal cells and reduced apoptosis. During early pregnancy, human decidua is composed of decidual cells, CD56 bright CD16‐d NK cells and macrophages.^30^ IL‐24 may affect early pregnancy by affecting early decidual stromal cells. Another report showed that IL‐24 inhibited normal vascular smooth muscle cells (VSMCs) growth by inhibiting the H_2_O_2_‐induced production of reactive oxygen species (ROS) and the expression of antioxidant enzymes, and VSMCs are the main actors of atherosclerosis.^31^ In this study, compared with the NRSA and NC groups, there were a significant increase in IL‐24 expression in PSRSA patients.

### 4.4 Ciliary neurotrophic factor

Ciliary neurotrophic factor (CNTF) belongs to the IL‐6 cytokine family^32^ and is involved in a variety of processes from endogenous neuroprotection to energy expenditure regulation in the body.^33^ Watanobe et al^34^ found that CTNF plays a role in the surge of steroid‐induced luteinizing hormone and prolactin in rat ovaries and believed that CNTF may be another body fluid connecting nutrition and reproductive function signalling. CNTF is related to energy metabolism, and strict control of glucose metabolism is essential for foetal growth and successful pregnancy outcomes. Abnormal insulin resistance is associated with preeclampsia. Akahori et al^35^ measured the levels of CNTF in non‐pregnant women, during normal pregnancy, post‐partum and in pregnancy with eclampsia and found decreased levels of CNTF in pregnant women compared with non‐pregnant women and a significant reduction in CNTF expression in eclampsia patients. In our study, there was a significant increase in CNTF expression in PSRSA patients and a decrease in CNTF expression in NRSA patients. The exact molecular mechanism for these controversial results remains unclear.

### 4.5 Eotaxin‐3

Shinkai et al^36^ first reported a new type of human CC chemokine with effective eosinophil activity in vascular endothelial cells stimulated by IL‐4 and named it eotaxin‐3 (also known as CCL26).^37^ There are multiple chemokines in the chemokine family that control the migration and invasion of extravillous trophoblasts (EVTs). Eotaxin‐3 has been confirmed to enhance EVT function and is closely related to thrombosis.^38^ Falcone et al^39^ showed that lower levels of serum eotaxin‐3 could be used as coronary heart artery independent predictors of future cardiovascular adverse events in patients. In the current study, we found that the serum eotaxin‐3 levels in PSRSA patients were significantly decreased compared with the NRSA and NC groups, consistent with previous reports. We hypothesize that low levels of eotaxin‐3 cause weakened EVT function and potential cardiovascular problems, which may cause recurrent spontaneous abortion in PSRSA patients.

### 4.6 B‐cell activating factor

B‐cell activating factor (BAFF), also known as TALL‐1, is an important growth factor for B cells.^40^ Guo et al^41^ found that the expression of BAFF in trophoblasts and decidua of women during normal, early pregnancy was higher than those in RSA patients at the same gestational time. Wei et al^42^ reported that decidual stromal cells express high levels of B‐cell lymphocyte activating factor receptor (BAFFR) early in a normal pregnancy. The combination of oestrogen and progesterone has a significant up‐regulation effect, suggesting that BAFFR may be involved in the regulation of embryo implantation and immunomodulation of the maternal‐foetal interface during early pregnancy. Our results indicated that the BAFF serum levels in PSRSA patients were significantly decreased compared to the NRSA and NC groups, suggesting that BAFF may play a role in the progression of PSRSA.

### 4.7 LAG‐3

Triebel et al^43^ first reported a novel lymphocyte activation gene closely related to CD4, belonging to the Ig superfamily, and named it LAG‐3. LAG‐3 inhibits T cell activation and cytokine secretion, thereby ensuring immune homeostasis.^44^ Compared with the control group, Zeng et al^45^ found that the expression level of LAG‐3 on the surface of CD14 + cells in peripheral blood mononuclear cells of RSA patients was down‐regulated, suggesting that their ability to present special antigens changed, which in turn promoted T cell activation involved in the pathogenesis of RSA. We obtained similar results. The serum level of LAG‐3 in PSRSA patients was obviously down‐regulated, compared with those in the NRSA and NC groups. The complete function of LAG‐3 in PSRSA patients requires further investigation.

## 5 CONCLUSIONS

In summary, DNAM‐1, IL‐24, CNTF, LAG‐3, BAFF, Eotaxin‐3 and EGF were identified as differentially expressed in PSRSA patients compared with the NRSA and NC groups. The present study had limitations, as the sample size was relatively small. A multi‐centre cohort of larger sample sizes of PSRSA and NRSA vs NC is needed to assess the diagnostic power of these seven biomarkers in the prethrombotic state of recurrent spontaneous abortion. Further research will be necessary to explore the related mechanisms between RSA and the prethrombotic state. The combination of protein array technology with the traditional prethrombotic state laboratory indicators and the endometrial blood perfusion assessment, and final follow‐ups of pregnancy outcomes will increase the accuracy, specificity and sensitivity of the diagnosis of RSA with prethrombotic state.

## CONFLICT OF INTEREST

The authors declare that no competing interests exist.

## AUTHOR CONTRIBUTIONS

**Ying Wu:** Conceptualization (equal); Data curation (lead); Funding acquisition (equal); Investigation (lead); Writing‐review & editing (lead). **Mingwei Xin:** Formal analysis (lead); Validation (lead). **Qian Han:** Data curation (equal); Software (lead). **Jingshang Wang:** Resources (lead). **Xiaodan Yin:** Formal analysis (equal). **Junqin He:** Project administration (lead). **Chenghong Yin:** Conceptualization (lead); Funding acquisition (lead); Supervision (lead); Writing‐review & editing (equal).

## Supporting information

Figure S1

Figure S2

Table S1

Table S2

Table S3

Table S4

## Data Availability

The data underlying this article are available in the article and in its Online [Link], [Link], [Link], [Link], [Link], [Link].
